# Wandering Accessory Spleen and Its Implications for Modern Clinical Practice

**DOI:** 10.3390/jcm14113901

**Published:** 2025-06-01

**Authors:** Agata Grochowska, Bartosz Marek Czyżewski, Karol Kamil Kłosiński, Piotr Tomasz Arkuszewski

**Affiliations:** 1 Students’ Scientific Association, Department of Biomedicine and Experimental Surgery, Medical University of Łódź, Narutowicza 60, 90-136 Łódź, Poland; agata.grochowska@stud.umed.lodz.pl (A.G.); bartosz.czyzewski@stud.umed.lodz.pl (B.M.C.); 2 Department of Biomedicine and Experimental Surgery, Faculty of Medicine, Medical University of Łódź, Narutowicza 60, 90-136 Łódź, Poland; karol.klosinski@umed.lodz.pl

**Keywords:** wandering accessory spleen, wandering spleen, accessory spleen, abdominal surgery, ectopic abdominal mass, splenic anomalies, abdominal pain

## Abstract

**Background:** A wandering accessory spleen (WAS) is defined as an ectopic accessory spleen with a long vascular pedicle characterized by marked anatomical mobility. “Wandering” can be congenital or acquired due to splenomegaly or pregnancy. The study aimed to analyze the clinical course of WAS and its symptoms. **Methods**: The desk research method and statistical analysis were used to assess the outcome. **Results**: In total, 12 cases of WAS managed operatively, including 11 women and 1 man, have been found. The correct WAS diagnosis was established before surgery in 3 of the 12 patients. One individual did not exhibit any symptoms and underwent surgery to avoid WAS torsion and infarction. Abdominal pain was the most commonly reported symptom in 11 patients. No mortality has been reported. **Conclusions**: WAS is a rare anomaly. It can be symptomatic or produce a spectrum of symptoms, such as abdominal pain, and may resemble abdominal neoplasms. Torsion is the most common pathology in the WAS study group and is most common in young people.

## 1. Introduction

A wandering spleen is a rare entity resulting from abnormal ligamentous laxity that fails to fixate the spleen in its usual location in the left upper quadrant, either congenitally or acquired due to conditions like splenomegaly or pregnancy. It leads to splenic hypermobility, an elongated vascular pedicle prone to torsion, and splenic infarction. Wandering spleen is more common in women of reproductive age and children, with congenital anomalies likely playing a significant role [1,2,3]. It can be asymptomatic or can cause mild to severe abdominal pain, gastric outlet obstruction, or symptoms mimicking acute pancreatitis [4,5].

An accessory spleen (AS) is a congenital anomaly characterized by splenic tissue forming as separate nodules distinct from the primary spleen [6,7,8]. Its prevalence varies and is estimated to be about 10–30%, depending on the study and group location, peaking in North America and Europe. In autopsies, it is over 10% [9]. In laparotomies, its prevalence is about 15% [10].

A wandering accessory spleen (WAS) is defined as an ectopic accessory spleen with a long vascular pedicle characterized by marked anatomical mobility [11,12]. Its presence can be non-symptomatic and diagnosed accidentally as part of the radiological diagnostic process. WAS can manifest itself as an acute abdomen, but can also cause recurrent pain caused by pressure on the internal organs. It can manifest itself in torsion, infarction, and rupture; however, these are complications and are not required to occur for a diagnosis of WAS.

In the scientific data, few cases have been described as wandering accessory spleen (WAS), and these are only reports of single cases [12,13,14,15,16,17,18,19,20,21,22,23,24,25]. WAS is a pathology that is not reported on a common basis; thus, the scientific data are lacking. A comparative analysis of the clinical course of WAS is lacking in the literature; hence, the authors’ objective is to analyze the clinical course of this anatomical peculiarity in correlation with the symptoms caused by a wandering primary spleen, which is the aim of the study.

## 2. Materials and Methods

The materials comprised 12 cases of a WAS published between 1965 and 2024 (only complete cases, not abstracts). The desk research method was used, involving existing data described as individual cases by other researchers. The research was monographic, qualitative, and quantitative in nature, conducted in the form of a detailed description. Separate cases were obtained after searching the following Internet databases: PubMed, ClinicalKey, Academic Search Ultimate (EBSCO), BMJ Journals, Elsevier Journals, Embase, Karger, Oxford Journals, Scopus, Springer, and Wiley Online Library. Google searches found some articles. All searches were conducted between April and December 2024. The search strategy was the following: (wandering OR dislocated OR misplaced) AND (accessory OR additional OR supernumerary) AND spleen *. The results were the following: PubMed-61; EMBASE-86 results. Those results were checked for duplicates, and duplicates were removed before screening. A systematic search using those databases with duplicate removal helped to find 10 articles that were included in the analysis. Due to a small number of found cases, Google Scholar was also checked for non-indexed literature, helping to find 2 articles that were included in the analysis. The study group included only cases in which the authors themselves used the term WAS to name the abdominal pathology they described. Only surgically treated cases were analyzed and not based solely on radiological observation, as intraoperative evaluation, often supported by histopathological examination, leaves no doubt about the nature of the lesion.

Each WAS case was entered into a case card; the data included patient age, sex, presence of torsion, medical history, preoperative diagnosis, treatment, pathology, size, and location of the WAS.

The results were also subjected to a basic statistical analysis. Arithmetic means, standard deviations, and minimum and maximum values were calculated briefly.

## 3. Results

The findings are presented in Table 1.

In total, 12 wandering accessory spleen (WAS) cases were found, including 11 women and 1 man. The ages of the patients ranged from 10 months to 46 years. The mean age was 17.9 ± 12.1 years, and half of the subjects were no older than 15.5 years. In all cases, WAS was found in the abdominal or pelvic cavity.

In all cases of WAS, surgical treatment was performed with splenectomy by laparotomy (eight cases) and laparoscopy (four cases). The final surgical diagnosis was WAS torsion in seven instances and uncomplicated WAS in four cases. WAS caused bowel obstruction in one case.

Abdominal pain was the most notable complaint in 11 cases, and fever was noted in 4 cases. Nausea or vomiting was observed in six cases. Intestinal obstruction signs were reported in one case. A movable abdominal mass was reported in three cases. Other rarer complaints included abdominal tenderness, bloating, lethargy, elevation of indicators of inflammation, occasional dysuria, and diarrhea. One patient felt no symptoms, as the WAS was an incidental finding at a pelvic ultrasound performed for other reasons.

The main indication for surgery was a syndrome of various symptoms that ultimately turned out to be due to torsion of WAS, which occurred in 7 out of 12 cases. Other indications for surgical intervention included:

- Intestinal obstruction due to external pressure on the intestine by WAS (initially suspected to be caused by adhesions, a mass in the right pelvis—a right ovarian cyst, a right hemosalpinx, or an extrauterine pregnancy)-1 case
- Symptomatic intraperitoneal mass (considered to be a subserous myoma, ectopic spleen, or atypical ovarian)-1 case
- Small subcapsular hematoma on the WAS and the risk of WAS torsion and infarction-1 case
- Prevention of the risk of WAS torsion-1 case
- Suspicion of an intraperitoneal gastrointestinal stromal tumor (GIST)-1 case

No mortality has been reported.

Only one patient was asymptomatic and was operated on to prevent torsion or infarction of WAS. The proper diagnosis of WAS was made before surgery in 3 out of 12 patients.

In three cases, the patient had more than one accessory spleen. In one of them, multiple WAS and AS were identified (three wandering accessory spleens were found, with an additional three accessory spleens). In two other cases, WAS was accompanied by two accessory spleens in one instance and a single accessory spleen in another.

## 4. Discussion

The accessory spleen is generally a common anatomical variant of the spleen. However, WAS is almost nowhere to be found in the scientific data. There is no article where authors describe more than one of their instances of WAS; all cases found were singular patients. This fact demonstrates the extreme rarity of this pathology.

In the study, a few main groups of patients can be distinguished. The first and most common group included patients who underwent surgery due to acute symptoms, primarily resulting from WAS torsion or infarction (seven cases), as well as one case of bowel obstruction caused by WAS. The second group involved patients who were operated on mainly due to recurrent abdominal pain resulting from pressure on abdominal structures, the presence of an unspecified abdominal or pelvic mass, and as a preventive measure (three patients). The third group consisted of asymptomatic individuals in whom WAS was found incidentally during radiological diagnostics performed for unrelated reasons (one case). These categories illustrate the variability in clinical presentation and the importance of individualized diagnostics.

Torsion and the associated symptoms are standard features of the clinical presentation of WAS, but they are not required for the diagnosis. Torsion of WAS indicates surgery and seems difficult to diagnose preoperatively. The diagnosis is usually made intraoperatively or postoperatively, where a pathologist can assess the specimen under a microscope or a surgeon can visually evaluate the WAS presence during surgery. For this uncommon medical condition, laparotomy was dominant over laparoscopy (eight vs. four). Technical difficulties, anatomical inconveniences, and emergency reasons might cause the preference.

In the scientific data, Kuroiwa et al. described a case of a 20-year-old female with no specific medical history with abdominal pain treated conservatively. However, a CT scan was performed, and the accessory spleen torsion was concluded. As for the mild symptoms, she was managed conservatively at first. Two months later, the patient underwent CT again, revealing that the accessory spleen torsion had resolved independently. Considering the risk of possible re-torsion and bleeding, the patient was referred for surgery. Postoperatively, it was confirmed that the artery on the infarcted focus side had evidence of occlusion and reopening [26]. Regarding all the possible reasons, in every suspected recurrent pain caused by an AS or WAS, surgery should be proposed [16,27].

Torsion of the vascular pedicle of the AS can also be caused by blunt trauma. Yoshida et al. described a case of a 12-year-old male presenting with left-sided abdominal pain after being beaten in the area. A CT scan and ultrasound revealed a 4 cm in diameter oval mass in the upper left abdomen that was resected 25 days after the injury. It was confirmed to be an AS adhered to the omentum and colon, twisted four times around its axis. Pathological examination revealed hemorrhagic infarction of the accessory spleen, confirming the diagnosis of accessory spleen torsion [28]. Despite this, the authors did not consider this case to be a WAS, perhaps because the torsion of the AS showed an apparent temporal relationship with blunt abdominal trauma and was, therefore, not a spontaneous torsion.

Apart from torsion, AS, including WAS, can cause various abdominal symptoms. It can cause spontaneous rupture with bleeding [29,30]. Patients who have not expressed WAS torsion symptoms were operated on mainly due to suspicion of neoplasm, recurrent abdominal symptoms, and risk prevention of WAS torsion [13,15,16,18,23]. In cases of pregnancy or splenomegaly, intestinal obstruction of WAS can occur. The only case of intestinal obstruction was caused by a big WAS (14 × 13 × 12 cm) and pregnancy, at the same time compressing the adjacent sigmoid colon and the small intestine [13]. When total splenectomy is considered for ITP or hematological reasons, every AS, including WAS, should also be found and removed during surgery. A thorough inspection of the abdominal cavity should be conducted [31,32].

The complications and symptoms of WAS in our study (recurrent abdominal pain, tenderness, nausea, vomiting, obstipation, intestinal obstruction, dysuria, and torsion) do not differ from those of wandering spleen [4,5,33,34,35].

The prognosis of WAS is favorable, especially when diagnosed early and treated surgically. All reviewed cases resulted in full recovery without mortality. Timely intervention helps prevent complications such as torsion or infarction.

The analysis is limited by the small number of available cases and reliance on published reports, which may introduce selection bias. The rarity of WAS hinders broader conclusions and limits statistical analysis. Additional limitations include the retrospective nature of the data and the limited standardized outcome measures across cases.

## 5. Conclusions

1. The wandering accessory spleen is a rarely reported clinical condition.
2. WAS can be asymptomatic or can cause a broad spectrum of symptoms. In most cases, torsed WAS was causing acute abdominal symptoms and ischemic changes, indicating the necessity for surgery.
3. The symptoms of WAS do not differ from those of a wandering spleen.
4. Due to its atypical anatomical position, a WAS may be misdiagnosed as an abdominal neoplasm. Nevertheless, single abdominal neoplasms are indications for surgery; therefore, identifying the indications for surgery is less challenging.
5. If the surgery is for hematological reasons, it is essential to remember that the accessory spleen, including WAS, can be multiplied, and a thorough examination of the abdominal cavity should be conducted.

## Figures and Tables

**Table 1:** All the analyzed cases. [The full table can be found in the Supplementary Materials, Table S1].

Source of Information	Symptoms Caused by WAS and Medical History	Preoperative Diagnosis of WAS	Method of Surgery	Dimensions of WAS [cm]	The Last Established Location of WAS	Final Diagnosis
Clifford, 1965 [13]	Intestinal obstruction (abdominal distention, nausea, vomiting, obstipation, crampy, colicky abdominal pains for five days); pregnancy	-	Laparotomy, splenectomy of WAS	14 × 13 × 12	Left lower quadrant of the pelvis, extending laterally, upwards up to about the level of the umbilicus, and downwards in the region of the posterior cul-de-sac	Bowel obstruction caused by WAS
Valls, 1998 [14]	Left upper abdominal and left lumbar pain, nausea, fever; 2-year history of diffuse self-limited abdominal pain; tenderness in the left upper quadrant without rebound	-	Laparotomy, splenectomy of WAS	6	Adjacent to the pancreatic tail (caudal portion of the pancreas) and below the lower pole of the left kidney	Torsion of WAS
Vural, 1999 [15]	Recurrent dull pain in the left lower quadrant of the abdomen; palpable mobile mass freely movable by approx. 7 cm in all directions	-	Laparotomy, splenectomy of WAS	4.5 × 4	Left lower quadrant of the abdomen, near the uterus, left-sided, intraperitoneal	WAS manifesting as an intraperitoneal mass
Kaniklides, 1999 [16]	Recurrent intermittent abdominal pain and occasional dysuria; a bicycle accident several months before presentation (at follow-up, 3 months later, she had no symptoms); a small subcapsular hematoma on WAS and 2 other accessory spleens	+	Laparotomy, splenectomy of WAS due to a risk of torsion and infarction (2 accessory spleens left intact)	8 × 15 (removed WAS)	Very close to the orthotopic spleen	WAS with 2 accessory spleens
Tandilava, 2014 [17]	Acute abdominal pain, repeated vomiting, and low-grade fever for about 48 h; the abdomen painful on palpation in the lower half, especially in the right iliac region and above the pubis, where a hard, painful formation was identified; tension in the muscles of the anterior abdominal wall, weakly positive symptoms of peritoneal irritation	-	Laparotomy, splenectomy of 3 wandering accessory spleens in the right pelvic area (3 accessory spleens in the upper left area left intact)	7.76 × 5.21 × 5.28 (the main WAS and two smaller wandering accessory spleens)	Right pelvic region (removed wandering accessory spleens); left upper area (3 remaining accessory spleens)	Torsion of 3 wandering accessory spleens on one twisted pedicle out of 6 accessory spleens
Perin, 2014 [18]	No specific symptoms caused by WAS	+	Laparoscopy, splenectomy of WAS (surgery because of the potential risks resulting from torsion or infarction of the WAS)	6 × 5	The pelvic cavity, near the left ovary	WAS
Termos, 2017 [19]	A few hours’ history of severe diffuse abdominal pain, mainly in the left upper quadrant (sudden in onset, aching, radiating to the left intra-scapular area and left shoulder), associated with nausea and 3 episodes of non-bilious and non-bloody vomiting; marked upper abdominal tenderness mainly over the epigastric area and left hypochondrium with voluntary guarding	-	Laparotomy, splenectomy of WAS due to torsion (accessory spleen near the native spleen left intact)	13 × 6 × 3.2	Left upper quadrant (WAS); near the native spleen (AS)	Torsion of WAS, accessory spleen
Mustafa, 2021 [20]	Severe abdominal pain for 5 days, nausea, episodes of non-bilious vomiting, moderate pyrexia (38.5 °C); marked lower abdominal tenderness mainly over the umbilical area and left lower quadrant, with signs of peritoneal irritation; a solid mass in the left of the umbilicus detected by palpation, elevation of indicators of inflammation	-	Laparotomy, splenectomy of WAS	5 × 5 × 5	Left adnexal area	Torsion of WAS
Wang, 2022 [21]	Irritability, fever for 5 days	+	Laparotomy, splenectomy of WAS	8 × 5 × 3	Left flank	Torsion of WAS
Sokolov, 2023 [22]	Signs of an acute intestinal infection; lethargy, repeated vomiting, restlessness, diarrhea for two days; splenectomy due to torsion and necrosis of the wandering spleen 2 months ago	-	Laparoscopy, splenectomy of WAS	4 × 3 × 3	Left subdiaphragmatic space	Torsion of WAS
Ferrer-Inaebnit, 2023 [23]	Intermittent colicky abdominal pain; intraperitoneal tumor in CT oriented as a gastrointestinal stromal tumor (GIST)	-	Exploratory laparoscopy, splenectomy of WAS	3.2 × 3.2 × 3.4	Intraperitoneal left posterior abdominal void	WAS
Locurto, 2024 [12]	Sudden upper abdominal pain, hypotension; abdominal tenderness mainly in the right flank and upper abdomen with mild peritoneal signs of rebound and guarding, a palpable mass in the periumbilical area, remarkable abdominal bloating, torpid peristalsis on auscultation; two months earlier, diagnosed with membranous glomerulonephritis and signs of nephrotic syndrome	-	Emergency exploratory laparoscopy, splenectomy of WAS	6.9 × 4.8 × 5.3	Variable position: left abdomen (in correspondence of the mesentery, close to the left rectus abdominis muscle) on CT; right upper abdomen (in front of the right kidney, close to the right rectus abdominis muscle, with coarse vascular pedicle) on MRI; close the transverse mesocolon during the surgery	Torsion of WAS

## Data Availability

The original contributions presented in this study are included in the article/Supplementary Materials. Further inquiries can be directed to the corresponding author.

## Supplementary Materials

The following supporting information can be downloaded at: https://www.mdpi.com/article/10.3390/jcm14113901/s1, Table S1: All the analyzed cases.
