# Supplementary material for: Wandering Accessory Spleen and Its Implications for Modern Clinical Practice
Source: J Clin Med. 2025 Jun 1;14(11):3901. doi: 10.3390/jcm14113901 (PMC12155822; doi:10.3390/jcm14113901)
Supplement: Supplementary file 1 [file jcm-14-03901-s001.zip › jcm-3636518-supplementary.pdf]

Table S1. All the analyzed cases.

Source of information	Age	Sex	Symptoms caused by WAS and medical history	Preoperative diagnosis of WAS	Method of surgery	Dimensions of WAS [cm]	The last established location of WAS	Final diagnosis
Clifford, 1965 [13]	26	F	Intestinal obstruction (abdominal distention, nausea, vomiting, obstipation, crampy, colicky abdominal pains for five days); pregnancy	-	Laparotomy, splenectomy of WAS	14x13x12	Left lower quadrant of the pelvis, extending laterally, upwards up to about the level of the umbilicus and downwards in the region of the posterior cul-de-sac	Bowel obstruction caused by WAS
Valls, 1998 [14]	13	F	Left upper abdominal and left lumbar pain, nausea, fever; 2-year history of diffuse self-limited abdominal pain; tenderness in the left upper quadrant without rebound	-	Laparotomy, splenectomy of WAS	6	Adjacent to the pancreatic tail and below the lower pole of the left kidney	Torsion of WAS
Vural, 1999 [15]	26	F	Recurrent dull pain in the left lower quadrant of the abdomen; palpable mobile mass freely movable by approx. 7 cm in all directions	-	Laparotomy, splenectomy of WAS	4.5x4	Left lower quadrant of the abdomen, near the uterus, left-sided, intraperitoneal	WAS manifesting as intraperitoneal mass
Kaniklides, 1999 [16]	5	F	Recurrent intermittent abdominal pain and occasional dysuria; a bicycle accident several months before presentation (at follow-up, 3 months later, she had no symptoms); a small subcapsular hematoma on WAS and 2 another accessory spleens	+	Laparotomy, splenectomy of WAS due to a risk of torsion and infarction (2 accessory spleens left intact)	8x15 (removed WAS)	Very close to the orthotopic spleen	WAS with 2 accessory spleens
Tandilava, 2014 [17]	14	F	Acute abdominal pain, repeated vomiting, and low-grade fever for about 48 hour; the abdomen painful on palpation in the lower half, especially in the right iliac region and above the pubis, where a hard, painful formation was identified; tension in the muscles of the anterior abdominal wall, weakly positive symptoms of peritoneal irritation	-	Laparotomy, splenectomy of 3 wandering accessory spleens in the right pelvic area (3 accessory spleens in the upper left area left intact)	7.76×5.21 ×5.28 (the main WAS and two smaller wandering accessory spleens)	Right pelvic region (removed wandering accessory spleens); left upper area (3 remaining accessory spleens)	Torsion of 3 wandering accessory spleens on one twisted pedicle out of 6 accessory spleens
Perin, 2014 [18]	17	F	No specific symptoms caused by WAS	+	Laparoscopy, splenectomy of WAS (surgery because of the potential risks resulting from a	6x5	Pelvic cavity, near the left ovary	WAS

					torsion or infarction of the WAS)			
Termos, 2017 [19]	27	F	Few hours history of severe diffuse abdominal pain, mainly in the left upper quadrant (sudden in onset, aching, radiating to the left intra-scapular area and left shoulder), associated with nausea and 3 episodes of non-bilious and non-bloody vomiting; marked upper abdominal tenderness mainly over the epigastric area and left hypochondrium with voluntary guarding	-	Laparotomy, splenectomy of WAS due to torsion (accessory spleen near the native spleen left intact)	13×6x3.2	Left upper quadrant (WAS); near the native spleen (AS)	Torsion of WAS, accessory spleen
Mustafa, 2021 [20]	12	F	Severe abdominal pain for 5 days, nausea, episodes of non-bilious vomiting, moderate pyrexia (38.5°C); marked lower abdominal tenderness mainly over the umbilical area and left lower quadrant, with signs of peritoneal irritation; a solid mass in the left of umbilicus detected by palpation, elevation of indicators of inflammation	-	Laparotomy, splenectomy of WAS	5×5x5	Left adnexal area	Torsion of WAS
Wang, 2022 [21]	9	M	Irritability, fever for 5 days	+	Laparotomy, splenectomy of WAS	8x5x3	Left flank	Torsion of WAS
Sokolov, 2023 [22]	10 month	F	Signs of an acute intestinal infection; lethargy, repeated vomiting, restlessness, diarrhea for two days; splenectomy due to torsion and necrosis of the wandering spleen 2 months ago	-	Laparoscopy, splenectomy of WAS	4x3x3	Left subdiaphragmatic space	Torsion of WAS
Ferrer-Inaebnit, 2023 [23]	46	F	Intermittent colicky abdominal pain; intraperitoneal tumor in CT oriented as a gastrointestinal stromal tumor (GIST)	-	Exploratory laparoscopy, splenectomy of WAS	3.2x3.2x3.4	Intraperitoneal left posterior abdominal void	WAS
Locurto, 2024 [12]	19	F	Sudden upper abdominal pain, hypotension; abdominal tenderness mainly in the right flank and upper abdomen with mild peritoneal signs of rebound and guarding, a palpable mass in the periumbilical area, remarkable abdominal bloating, torpid peristalsis on auscultation; two months earlier,	-	Emergency exploratory laparoscopy, splenectomy of WAS	6.9×4.8×5.3	Variable position: left abdomen (in correspondence of the mesentery, close to the left rectus abdominis muscle) on CT; right upper abdomen (in front of the right kidney, close the right rectus abdominis muscle, with coarse vascular pedicle) on MRI;	Torsion of WAS

			diagnosed with membranous glomerulonephritis and signs of nephrotic syndrome				close the transverse mesocolon during the surgery	
